# Impact of Change in Lifestyle and Exercise on Cognitive Function in Patients With Rheumatoid Arthritis: A Systematic Review

**DOI:** 10.7759/cureus.18268

**Published:** 2021-09-25

**Authors:** Aqsa Akram, Petros Georgiou, Wangpan Shi, Matthew C Proute, Tatsiana Serhiyenia, Roshini Pradeep, Mina E Kerolos, Nageshwar Kothur, Safeera Khan

**Affiliations:** 1 Internal Medicine, California Institute of Behavioral Neurosciences & Psychology, Fairfield, USA; 2 Pathology, California Institute of Behavioral Neurosciences and Psychology, Fairfield, USA; 3 General Medicine, California Institute of Behavioral Neurosciences & Psychology, Fairfield, USA

**Keywords:** physical exercise, cognitive dysfunctions, cognitive performance, rheumatology & autoimmune diseases, life style

## Abstract

Rheumatoid arthritis (RA) is a chronic destructive type of arthritis. It has a high prevalence in females as compared to the male population globally. It mainly affects the synovium of peripheral joints and leads to the destruction of joints with time. Patients with RA usually have a high burden of inflammation which may lead to certain physical disabilities and debilitating effects on mental health and cognitive ability. The question we investigated here in this systematic review is how changing lifestyles and increasing exercise or physical activities affects one's cognitive abilities. This article adheres to preferred reporting items for systematic review and meta-analyses (PRISMA) guidelines. We used different databases such as PubMed, MEDLINE, and ScienceDirect to find relevant articles. To ensure the quality of the finally selected 12 studies, we followed different quality appraisal tools.

Based on our review, we found out that increasing physical activities and aerobic exercises positively increase overall well-being and decrease the inflammatory load, which will ultimately positively impact cognitive function in this subgroup of patients. We also discover certain key players affecting motivation, perception, and adherence to physical activities. We encourage future studies to be done on this topic to help in increasing quality of life and increasing independence in this group of patients. Counseling and addressing patient concerns are very important and keep disease activity well controlled so that physical activities become feasible.

## Introduction and background

The worldwide prevalence of rheumatoid arthritis (RA) was estimated as 0.24 percent based upon the Global Burden of Disease 2010 Study [1]. The prevalence of RA in the US has increased; it has been estimated that about 1.28-1.36 million adults were diagnosed in 2014 [2]. The risk of developing RA is 3.6 percent in women and 1.7 percent in men [3]. In other words, one in every 12 women and one in every 20 men are predicted to develop an autoimmune rheumatic disease [3].

Rheumatoid arthritis (RA) is a type of inflammatory arthritis that primarily affects peripheral joints' synovium [4]. It is the most common inflammatory arthropathy globally, which leads to joint destruction and the inability to perform functions properly. RA is diagnosed by the presence of anti-citrulline protein antibodies (anti-ccp) and rheumatoid factor (RF) in the serum [5]. Chronic inflammatory disorders such as RA has a higher prevalence of cognitive disabilities than the general population [6]. Cognitive ability is described as one's ability to manage their daily tasks, plan their days, ability to maintain concentration, overall performance, and decision-making capacity, as well as memory retaining capacity [7]. Therefore, intact cognitive function is very crucial, particularly for a patient with rheumatoid arthritis. Cognitive function is thought to be linked with chronic inflammation and pain. One study hypothesized that neurons involved in processing cognitive abilities are also closely involved in the pain responses; they may have a reciprocal effect on one another [7]. A review study in RA patients showed that patients with chronic RA underperform in cognitive ability tests compared to the healthy general population [8].

Many studies on this topic reported that cognitive function is also affected by RA. Bartolini et al. carried out a psychometric assessment of several patients with RA exploring several cognitive aspects and found out that cognitive disability was common in RA patients [8]. They concluded that a scission between the parietal-frontal lobe and subcortical white matter due to microangiopathy; furthermore, a chronic reduction in sensory stimuli by destructed joints could lead to an amendment in cognitive planning [8]. Although many kinds of research have been conducted on the relationship between exercise and cognitive function in RA, very little is known about its impact on lifestyle, exercise, and physical activities on improving the cognitive ability of patients with RA. Literature has shown that cognitive inabilities are compromised in about 38% to 71% of individuals with RA [8]. Our study aims to systematically review the documented literature on this topic from a different dimension to insight into the effects of the changing lifestyle, increasing exercise, and its positive effects on cognitive performance in patients with RA.

## Review

In this section, we will discuss how we collected our data and reviewed it systematically. This review analyzes the impact of a healthy lifestyle and exercise in improving cognitive disabilities in patients with chronic inflammatory disorders such as RA.

Method

Our study complies with the Preferred Reporting Items for Systematic Reviews and Meta-Analyses (PRISMA) protocol [9].

Search Strategy

We searched multiple electronic databases, such as PubMed, MEDLINE, ScienceDirect, PubMed Central, to extract our data. We used the medical subject headings (MeSH) terms and keywords individually and identified relevant articles. The MeSH terms and keywords used in the search strategy were "exercise," "physical activity," "aerobic exercises," "rheumatoid arthritis," "cognitive ability, "prevention and control," and" therapeutic/exercise'. The summary of MeSH terms used as search strategy is exhibit in Table 1.

**Table 1:** Summary of MeSH search strategy. MeSH: medical subject headings.

MeSH term	Total articles on PubMed
((“Exercise/prevention and control"[Majr] OR "Exercise/psychology"[Majr] OR "Exercise/therapeutic use"[Majr])) AND ((“Arthritis, Rheumatoid/diet therapy"[Majr] OR "Arthritis, Rheumatoid/prevention and control"[Majr] OR "Arthritis, Rheumatoid/psychology"[Majr] OR "Arthritis, Rheumatoid/therapy"[Majr]))	Total 28 articles

The screening of articles was done by going through the topic's name and abstracts and keeping the ones relevant to our research question. Inclusion and exclusion criteria were strictly followed to screen the relevant studies only. After the screening, articles were checked for quality appraisal, based on the type of studies, using different quality appraisal tools such as the Joanna Briggs Institute (JBI) quality appraisal checklist, Cochrane checklist, and Scale for the Assessment of Narrative Review Articles (SANRA) checklist. In our review, we have included mixed types of studies.

Inclusion Criteria

For our study selection, we included the following criteria: studies conducted in the English language, only adult human population studies, articles published in the last 10 years relevant to our topic and research question, peer-reviewed, full texts. We included randomized clinical trials (RCTs) and observation studies i.e., observational cohort studies, cross-sectional studies, and literature reviews.

Exclusion Criteria

Exclusion criteria for our study is grey literature, books, unpublished papers, letter to editor, editorials, duplicate and overlapping studies, in vitro or animal studies, studies conducted on children below eighteen, non-full text, not peer-reviewed, papers published more than 10 years ago, and articles in other languages.

Results

**Figure 1:**
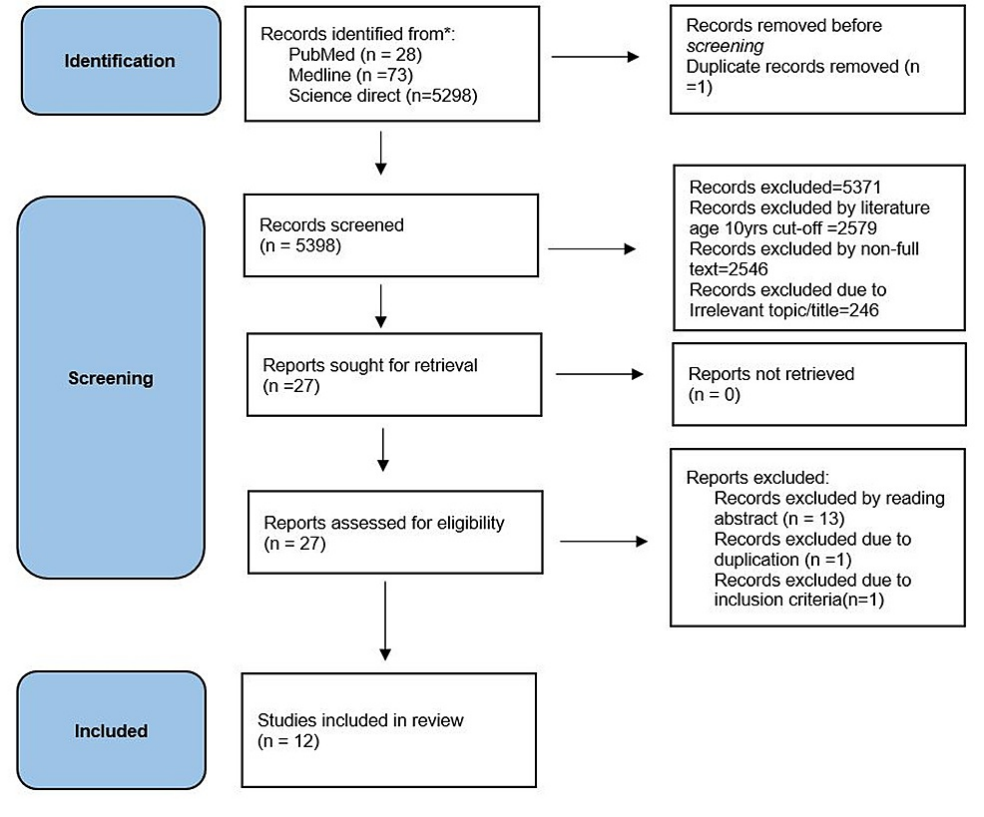
PRISMA flow diagram. PRISMA, Preferred Reporting Items for Systematic Reviews and Meta-Analyses [9].

A total of 5399 articles were recognized after using the MeSH strategy, other keywords, and topic titles on different research databases. Of these 5399, one paper was excluded as it was duplicated. The remaining 5398 articles were screened according to our inclusion criteria (full-text studies in English, last 10 years, on humans, clinical trials, all types of reviews, observational studies). Five thousand three hundred seventy-one published articles were dropped out at the initial screening stage due to topic and title irrelevance leaving 27 studies. Out of these 27 studies, we further screened them by reading the abstract and introduction to assessing for relevance to our research question and excluded one duplicate, one due to not meeting inclusion criteria, and 13 irrelevant studies. The final twelve articles were selected after screening and quality appraisal checklist and kept for our studies. Our study included mixed kinds of studies. We reviewed four observational cohort studies, two review articles, three cross-sectional studies, two randomized control studies (RCT), and one phenomenological study. Figure 1 shown below summarizes our whole screening process.

Table 2 outlines the summary of studies that were included in our study.

**Table 2:** Summary of studies. RA, rheumatoid arthritis; PA, physical activity; EE, energy expenditure; HEPA, health-enhancing physical activity; IA, inflammatory arthritis.

Author name	Year of publication	Purpose of study	Study Type	Result
Silva et al. [4].	2013	To look over the physical activity practice among patients with early RA and explore its possible association between utilitarian dysfunction, physical exercise, and disease activity.	Cross-sectional study	The study concluded that physical activities must be recommended to patients with RA.
Shadick et al. [6].	2019	To explore the clinical and utilitarian components related to a worsening of cognitive abilities in RA.	Cohort study	The prevalence of related cognitive issues was noticed less in RA patients who were more physically active.
Chaurasia et al. [7].	2020	Connect and pin down the frequency and different subgroups of cognitive dysfunction in Rheumatoid arthritis.	Review paper	Cognitive abilities were compromised in RA. But there are other factors as well which play a role in compromising these abilities negatively.
Larkin et al. [10].	2016	This study aimed to explore the acknowledgment of physical activities degree and psychological factors of physical activities among patients with inflammatory arthritis.	Cross-sectional study	This study reported that inflammatory arthritis (IA) has a lower practice of physical activity. Furthermore, their perception about PA is linked with the degree of self acknowledging PA and EE. This information provides protocols for laying out different interventions to tailor their insights about PA to increase physical activity practice in IA patients.
Nordgren et al. [11].	2012	The purpose of these guidelines is to draw the recruitment process, layout, appraisal methods, and the intervention program of a HEPA intervention study in RA patients.	Intervention study	This clinical intervention propose HEPA programs has befitting effects in some RA patients [12].
Pukˇsi'c et al. [13].	2021	To investigate the likelihood and potency of yoga in enhancing the psychological, physical, and health-related quality of life (HQOL) in patients with rheumatoid arthritis.	Randomized control study	This study reports no significant improvement in quality of life after implementing yoga in RA patient's life. However, they reported some positive effects in mood elevation. They also reported yoga was safe in this sub-group of the population.
Hegarty et al. [14].	2015	This study was conducted to explore the role of physical activities in moderating the relationship between mood and fatigue in RA.	Cohort study	It is documented that being highly physically active on the most fatigue day can positively affect the mood and decrease overall fatigue perception.
Lööf et al. [15].	2017	To understand and explore fear-avoidance perceptions regarding physical activity in patients with a high degree of rheumatic pain.	Phenomenological study	Their study reported that belief such as fear of provoking pain due to physical exercise undermines the perception of chronically ill patients. They also tend to perceive their body as physically fragile thereby, avoiding PA.
Rezaei et al. [16].	2014	This study was conducted to investigate the role of illness insight concerning its relationship amid pain and depression in people who have rheumatoid arthritis.	Cross-sectional study	The study reported (r = 0.57, p < 0.001) that depression symptoms were momentously associated with perceived pain.
Knittle et al. [17].	2016	This research investigated if a greater degree of self-governing motives, self productiveness for PA high-level use of self-regulation tools inter-pose in establishing PA and sustainability after six months of theory-based interviewer intervention and self-piloting interventional methods.	Randomized control trial	This study documented higher self-governing motives and self productiveness for PA following intervention interviewing positively in maintaining and establishing the PA.
Nordgren et al. [18].	2014	To appraise characteristics of constancy & its response during the first year of an outsourced 2-year HEPA program in patients with rheumatoid arthritis (RA).	Observational cohort study	This study observed a high constancy rate among RA patients. Overall health was significantly improved.
Salmon et al. [19].	2019	The purpose of this study was to come up with an intervention to assist in the self-regulation of fatigue caused by chronic RA by altering physical activity.	Review article	It reported the guidelines to draw theory-based intervention after consulting rheumatoid arthritis patients and clinicians to lower RA fatigue.

Discussion

Many chronic illnesses can negatively affect cognitive abilities; however, patients with RA are particularly at risk of developing it due to their complex medical treatment regimen [6]. However, according to many studies, the prevalence of cognitive impairment among individuals with inflammatory arthritis is higher than the general population [6]. Therefore, screening of neuropsychiatric issues in this subgroup is an important part of the treatment regimen. In this systematic review, we gathered twelve moderate-quality papers related to as many possible aspects of this disease to explore the impact of implementing regular exercises, PA, change in lifestyle on cognition function in the RA population.

Importance of Physical Activities in Inflammatory Arthritis

Physical activities play a crucial role in our life; previous studies have shown that it is recommended to perform at least 20 minutes of exercise per day in a week [4]. It has been documented in the past that cardiovascular diseases are the leading cause of death worldwide; patients with inflammatory arthritis such as RA are particularly at risk of such complications [10]. It is reported in an international study done among patients with RA in two years periods from 28 different cities of twenty-one countries documented only 13.8% of patients were physically active [4]. Physical activities such as running, walking, and jogging are proven to decrease this risk of cardiovascular complications among this sub-group of arthritis by reducing the pro-inflammatory leukotrienes such as Interleukin (IL)-6, C-reactive protein (CRP), tumor necrosis factor (TNF) alpha [6,10,11]. However, researchers have also found that cognitive problems in patients with RA are not secondary to carotid atherosclerotic changes; instead, it is due to high disease activity i.e., uncontrolled inflammation [6]. High levels of IL-6 and CRP are associated with an increased risk of developing early dementia [6]. As per treatment guidelines, physical activities should be included and routine medical therapies in RA [4]. However, some studies in the past have shown exceptions to this. Physical activities should be target related only to individual assessment of disease activity in each patient. For instance, intense exercise is only helpful in patients with well-controlled RA [4]. Yoga is another way of doing physical activity. It involves physical and breathing exercises which are linked to the autonomic nervous system and hypothalamic-pituitary plexus, which relaxes mood by releasing certain hormones. Literature reviews has shown the positive results of performing yoga in a patient with RA on overall well-being, quality of life, pain, and disease activity [13].

**Figure 2:**
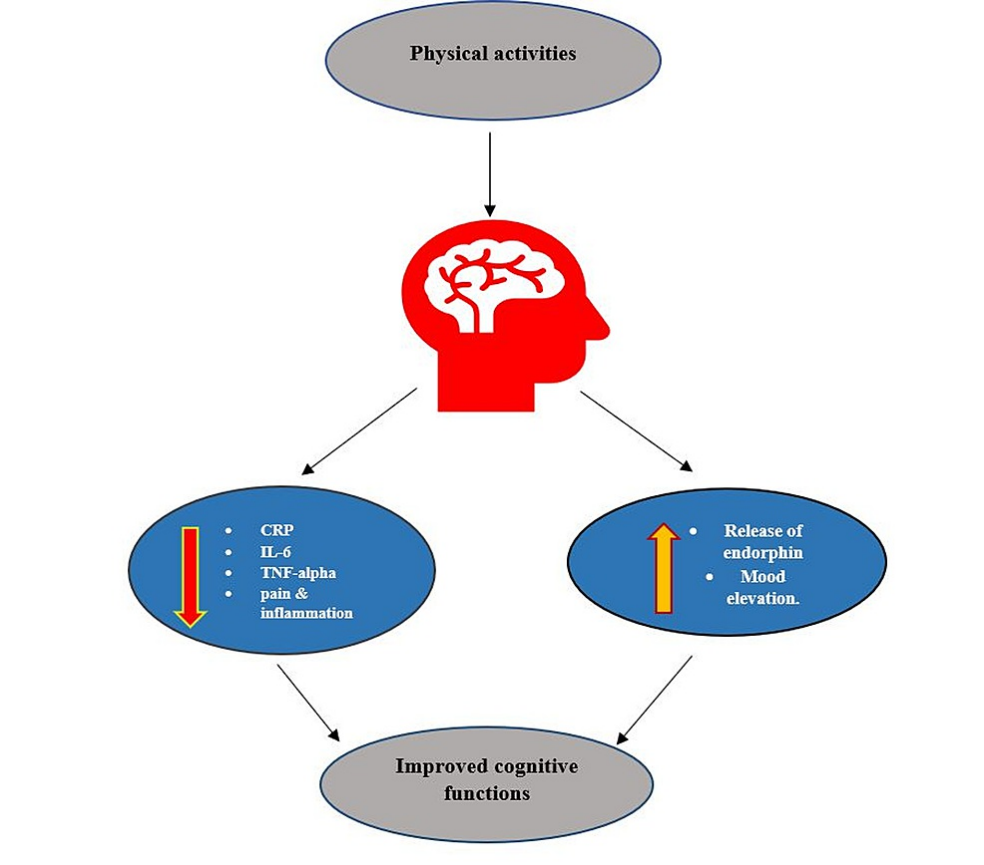
Relationship of physical activity and cognitive function. IL-6, interleukin 6; TNF-alpha, tumor necrosis factor alpha; CRP, C-reactive protein.

Previous research documented yoga as a safe and quite doable way of exercise in different musculoskeletal conditions, such as RA, osteoarthritis, and many more [13]. A randomized control trial (RCT) study concluded that yoga is proven to reduce psychological distress [13]. Physical activities are associated with releasing a hormone called endorphin which in return elevates the positive mood and reduces the negativity [14]. Therefore, increasing physical activity in patients with RA improves their mood and significantly improves their overall well-being and mental health [14]. A cohort study mentioned that if patients with RA meet the guidelines for physical activity, it will protect different domains of cognition such as protection against memory, word-finding, and concentration [6]. Figure 2 shown below is the nutshell effect of physical activities on a different aspect to overall increase cognitive function.

Factors Affecting the Ability to Perform Physical Activities in RA

Fear of pain provocation & cognitive function

Beliefs about physical activities vary among the various group of the population. For instance, patients with low back pain and rheumatic pain are afraid to start performing any aerobic exercise. It can provoke pain symptoms even more [10,15]. So, one factor that may contribute to refraining from physical activities and muscle-strengthening exercises in a patient with RA is fear of pain provocation. A study conducted in the past showed that targeting such beliefs by education and intervention in patients with rheumatoid arthritis can help them to become physically active again and will have long-term benefits despite short-term exacerbation of pain [10]. Another study reported that patients with chronic rheumatic pain have fear-avoidance belief towards physical activities and exercise [15]. As a result, addressing these beliefs in a regular setting can help this group of patients to have a more favorable attitude towards physical activities [15]. A cross-sectional study concluded that patients who suffer from persistent chronic pain rather than higher intensity pain would positively correlate with impaired cognition [20]. Their study also showed that persistent pain would pronouncedly compromise cognitive abilities compared to controls [20]. Patients with acute pain will show a pronounced compromise on psychomotor abilities [11].

Self-efficacy & belief

Another psychological factor that has a profound influence on physical activity (PA) is self-efficacy, best explained by a cross-sectional study done on 102 participants to judge one's capabilities to organize and perform certain actions [10]. According to a cross-sectional study done in the past, reported that physical disability that develops in the course of the chronic disease leads to disabilities in the social life and work life of patients as they fail to fulfill their chores all this leads to depression, and patients ultimately perceive the greater level of pain [16].

Depression in chronic inflammatory conditions

It is reported that about 14% to 62% diagnosed with RA suffer from depression [21]. Another important factor that may negatively affect physical activities in patients with RA is depression [16]. Since patients with RA suffer from different aspects of life, one of the most important is psycho-social; depression in this population will affect their moods and illness perception with a greater level of pain [16]. Cognitive-behavioral therapy effectively decreases the pain perception in people with RA [16]. It was concluded in a study that illness perception should be incorporated in the treatment of RA to improve the overall quality of life [16]. We hypothesize that if a patient's perception about illness is addressed in the right manner, it can decrease the pain perception and increase the ability to do physical activities.

Previous research has explored the effects of different exercises, physical activities, and yoga on overall well-being in patients with RA. According to a review article, cognitive abilities are compromised in patients with chronic pain. As per their study, cognition and pain processes are linked together; therefore, they can reciprocally modulate each other [7]. Previous studies done by different researchers found out that cognitive abilities are affected in patients with different kinds of inflammatory conditions [7]. However, another study found that pain was not the reason for cognitive disabilities in patients with RA when they put depression as the mediator between two variables [7].

Adherence to Physical Activities

Initiation of physical activities and maintaining them in patients with RA is quite challenging. A study documented that most patients with RA fail to maintain the PA; therefore, such patients need behavioral changes intervention to initiate and maintain the recommended PA [17]. A cohort study was conducted to explore the importance of adherence to physical activities. They observed that health-enhancing physical activity (HEPA), including 150 mins of exercise per week and moderate-intensity muscle-strengthening exercises twice a week in patients with RA, might be helpful along with treatment regimen [18]. Beliefs of perceived benefits of exercise perceive adherence to physical activities in patients with RA; therefore, the social support system will result in a high rate of exercise maintenance [11]. A randomized controlled trial was performed on patients with RA concluded that motivational interviews and self-regulation coaching could help patients to continue their PA [17]. According to an observational cohort study done by previous researchers on 220 participants with RA requiring participants to perform daily physical activities and twice-weekly circuit training along with support, meetings concluded that the Outstretched HEPA program has high retention and adherence rate is quite reasonable [18]. This study showed that the health outcomes were satisfactory; however, adherence to the program component relationship and response was unclear [18]. A study documented that support systems, circuit meetings, and expert lectures helped maintain the HEPA program among the RA population [11].

Limitations

Our study has some limitations since this is a systematic review, so we could not implement any previously suggested methods to improve cognition and their impact. This is a limited review of some selected moderate-quality papers. We might have missed several other important aspects of the study due to our inclusion/exclusion criteria.

## Conclusions

This systematic review aimed to determine the impact of change in lifestyle and increase in physical exercise on cognitive function in patients with rheumatoid arthritis. After reviewing published literature related to our research question we concluded several points, that patients with RA and other kinds of inflammatory arthritis have a high burden of inflammation, which is compromising their thought process and self-efficacy towards physical activity. We also extracted some data from previous studies on how pain perception and cognitive function are interconnected as well as pain and disease activity are interlinked. Therefore, patients with well-controlled disease activity i.e., joint inflammation can perform physical activities. Performing regular exercises decrease the risk of developing cognitive disabilities. But to maintain physical exercise as part of life is quite challenging. We encourage clinicians to closely monitor the disease activity and educate their patients about the impact of engaging in physical activities on different aspects of cognitive function as a part of routine clinic follow-ups.

## References

[1] Cross M, Smith E, Hoy D (2014). The global burden of rheumatoid arthritis: estimates from the global burden of disease 2010 study. Ann Rheum Dis.

[2] Hunter TM, Boytsov NN, Zhang X, Schroeder K, Michaud K, Araujo AB (2017). Prevalence of rheumatoid arthritis in the United States adult population in healthcare claims databases, 2004-2014. Rheumatol Int.

[3] Crowson CS, Matteson EL, Myasoedova E (2011). The lifetime risk of adult-onset rheumatoid arthritis and other inflammatory autoimmune rheumatic diseases. Arthritis Rheum.

[4] Silva CR, Costa TF, de Oliveira TT, Muniz LF, da Mota LM (2013). Physical activity among patients from the Brasília cohort of early rheumatoid arthritis. Rev Bras Reumatol.

[5] Chimenti MS, Fonti GL, Conigliaro P (2021). The burden of depressive disorders in musculoskeletal diseases: is there an association between mood and inflammation?. Ann Gen Psychiatry.

[6] Shadick NA, Katz P, Iannaccone CI, Maica G, Coblyn J, Weinblatt ME, Cui J (2019). The impact of exercise, lifestyle, and clinical factors on perceived cognitive function in patients with rheumatoid arthritis: results from a prospective cohort study. ACR Open Rheumatol.

[7] Chaurasia N, Singh A, Singh IL, Singh T, Tiwari T (2020). Cognitive dysfunction in patients of rheumatoid arthritis. J Family Med Prim Care.

[8] Bartolini M, Candela M, Brugni M (2002). Are behaviour and motor performances of rheumatoid arthritis patients influenced by subclinical cognitive impairments? A clinical and neuroimaging study. Clin Exp Rheumatol.

[9] Page MJ, McKenzie JE, Bossuyt PM (2021). The PRISMA 2020 statement: an updated guideline for reporting systematic reviews. BMJ.

[10] Larkin L, Gallagher S, Fraser AD, Kennedy N (2016). Relationship between self-efficacy, beliefs, and physical activity in inflammatory arthritis. Hong Kong Physiother J.

[11] Nordgren B, Fridén C, Demmelmaier I, Bergström G, Opava CH (2012). Long-term health-enhancing physical activity in rheumatoid arthritis--the PARA 2010 study. BMC Public Health.

[12] (2014). Health-enhancing physical activity in rheumatoid arthritis: prevalence, intervention and assessment. https://openarchive.ki.se/xmlui/handle/10616/42231.

[13] Pukšić S, Mitrović J, Čulo MI, Živković M, Orehovec B, Bobek D, Morović-Vergles J (2021). Effects of yoga in daily life program in rheumatoid arthritis: a randomized controlled trial. Complement Ther Med.

[14] Hegarty RS, Conner TS, Stebbings S, Treharne GJ (2015). Feel the fatigue and be active anyway: physical activity on high-fatigue days protects adults with arthritis from decrements in same-day positive mood. Arthritis Care Res (Hoboken).

[15] Lööf H, Johansson U (2018). “A body in transformation”—an empirical phenomenological study about fear‐avoidance beliefs towards physical activity among persons experiencing moderate‐to‐severe rheumatic pain. J Clin Nurs.

[16] Rezaei F, Neshat Doost HT, Molavi H, Abedi MR, Karimifar M (2014). Depression and pain in patients with rheumatoid arthritis: mediating role of illness perception. Egyptian Rheumatologist.

[17] Knittle K, De Gucht V, Hurkmans E, Vlieland TV, Maes S (2016). Explaining physical activity maintenance after a theory-based intervention among patients with rheumatoid arthritis: process evaluation of a randomized controlled trial. Arthritis Care Res (Hoboken).

[18] Nordgren B, Fridén C, Demmelmaier I, Bergström G, Lundberg IE, Dufour AB, Opava CH (2015). An outsourced health-enhancing physical activity programme for people with rheumatoid arthritis: exploration of adherence and response. Rheumatology (Oxford).

[19] Salmon VE, Hewlett S, Walsh NE, Kirwan JR, Morris M, Urban M, Cramp F (2019). Developing a group intervention to manage fatigue in rheumatoid arthritis through modifying physical activity. BMC Musculoskelet Disord.

[20] Gunnarsson H, Grahn B, Agerström J (2016). Impaired psychomotor ability and attention in patients with persistent pain: a cross-sectional comparative study. J Pain Res.

[21] Ziarko M, Siemiątkowska K, Sieński M, Samborski W, Samborska J, Mojs E (2019). Mental health and rheumatoid arthritis: toward understanding the emotional status of people with chronic disease. Biomed Res Int.

